# Four new orchid species from the Lengguru fold belt, West Papua, Indonesia

**DOI:** 10.3897/phytokeys.61.7590

**Published:** 2016-02-25

**Authors:** Lina Juswara, André Schuiteman, Vincent Droissart

**Affiliations:** 1 Herbarium Bogoriense, Botany Division, Research Center for Biology, Indonesian Institute of Sciences, Cibinong Science Centre, Jl. Raya Jakarta-Bogor Km 46, Cibinong-Bogor, 16911, Indonesia; 2 Herbarium, Royal Botanic Gardens, Kew, Richmond, Surrey, TW9 3AB, United Kingdom; 3 Institut de Recherche pour le Développement (IRD), Unité Mixte de Recherche AMAP (Botanique et Bioinformatique de l’Architecture des Plantes), Boulevard de la Lironde, TA A-51/PS2, F-34398 Montpellier Cedex 5, France; 4 Missouri Botanical Garden, Africa & Madagascar Department, P.O. Box 299, St. Louis, Missouri 63166-0299, U.S.A.; 5 Herbarium et Bibliothèque de Botanique africaine, CP 265, Université Libre de Bruxelles, Boulevard du Triomphe, B-1050, Brussels, Belgium

**Keywords:** *Bulbophyllum*, *Dendrobium*, Kaimana Regency, Kumawa Forest Reserve, limestone karst, Orchidaceae, *Taeniophyllum*, Triton Bay

## Abstract

*Bulbophyllum leucoglossum*, *Dendrobium centrosepalum*, *Dendrobium taeniocaule*, and *Taeniophyllum pyriforme* are here described as new species, based on herbarium specimens collected from the Lengguru fold-and-thrust belt in the West Papua Bird’s neck, Indonesian New Guinea. All four novelties were found growing in submontane forest (elevation > 1000 m a.s.l.) on limestone karst. Information concerning the distribution and habitat for these taxa is provided along with diagnostic features, line drawings, high resolution photographs, and a map of collecting localities. More field studies are required to find additional populations of these new species, in order to better characterize their habitat, ecology and conservation status.

## Abstract (id)

*Bulbophyllum leucoglossum*, *Dendrobium centrosepalum*, *Dendrobium taeniocaule*, dan *Taeniophyllum pyriforme* dideskripsikan sebagai jenis baru, dari spesimen herbarium yang dikoleksi dari beberapa lokasi di Lengguru, yang terletak di leher burung Papua Barat, Niugini-Indonesia. Empat jenis baru tersebut tumbuh di hutan submontana (ketinggian > 1000 mdpl) di wilayah tanah kapur. Informasi distribusi dan habitatnya disampaikan dalam tulisan ini lengkap dengan informasi karakter diagnosa, gambar pensil, foto, serta peta lokasi pengkoleksiannya. Penelitian lapangan selanjutannya masih diperlukan untuk mengetahui kondisi populasi jenis-jenis tersebut agar diperoleh informasi selengkapnya yang berhubungan dengan habitat, ekologi, dan status konservasinya.

## Abstract (fr)

*Bulbophyllum leucoglossum*, *Dendrobium centrosepalum*, *Dendrobium taeniocaule* et *Taeniophyllum pyriforme* sont décrits et illustrés, sur base de spécimens d’herbier collectés dans la zone de plissement et de chevauchement de Lengguru, dans la moitié occidentale indonésienne de l’île de Nouvelle-Guinée. Ces quatre nouveautés taxonomiques ont toutes été découvertes dans des forêts karstiques submontagnardes (altitude > 1000 m). Les informations concernant la distribution et l’habitat de ces espèces sont fournies avec leurs diagnoses, des dessins, des photographies à haute résolution, ainsi qu’une carte des localités inventoriées. Des études de terrain complémentaires sont nécessaires pour trouver des nouvelles populations pour ces espèces, afin de mieux caractériser leur habitat, leur écologie et leur statut de conservation.

## Introduction

Indonesia has been classified as a megadiverse country (Mittermeier et al. 1997), and is estimated to house approximately 10% (30,000 species) of the botanical diversity of the world (World Resources Institute et al. 2005). The country is composed of over thirteen thousand islands lining the equator. The largest island, New Guinea, is also known to harbour one of the richest orchid floras in the world, only surpassed by Colombia, Ecuador, and Peru (Schuiteman and de Vogel 2007). Ormerod (2014) recorded c. 2870 species of orchids from New Guinea, 11% of the world’s orchid flora, of which about 95 percent are endemic to the island (Schuiteman et al. 2001-2010). Considering that large parts of New Guinea still have low collecting densities, it is likely, as also suggested by Ormerod, that many species still await discovery here.

The island has a complex geological history, related to the tectonics of the Indo-Australian and Pacific plates. The Lengguru fold-and-thrust belt (Bailly et al. 2009) was formed by the collision of the Australian and Pacific plates, and is characterized by a series of parallel and oblique folds separated by deep valleys, which link the high mountains of the Central Range of western Papua to the moderately high mountains of the Bird’s neck. According to Vollering et al. (2016), the orchid species diversity in this part of New Guinea, which at present has a very low collecting density, is predicted to be relatively low, compared to the high diversity of the Eastern Highlands and Chimbu provinces of Papua New Guinea. This prediction was based on species distribution modelling using occurrence records of 532 species and spatial environmental data. It will be interesting to see if the prediction holds up once the area is better known botanically.

In October–November 2014, the first and the last authors took part in the Lengguru 2014 scientific expedition (www.lengguru.org). One of the main objectives of this multi-disciplinary research programme was to make a rapid but wide-ranging assessment of the botanical diversity in a major Papuan karst region. The ultimate goals are to generate species plant checklists for this poorly sampled area, and to incorporate these data into a regional database to further analyse plant distribution patterns at different scales.

**Figure 1.**
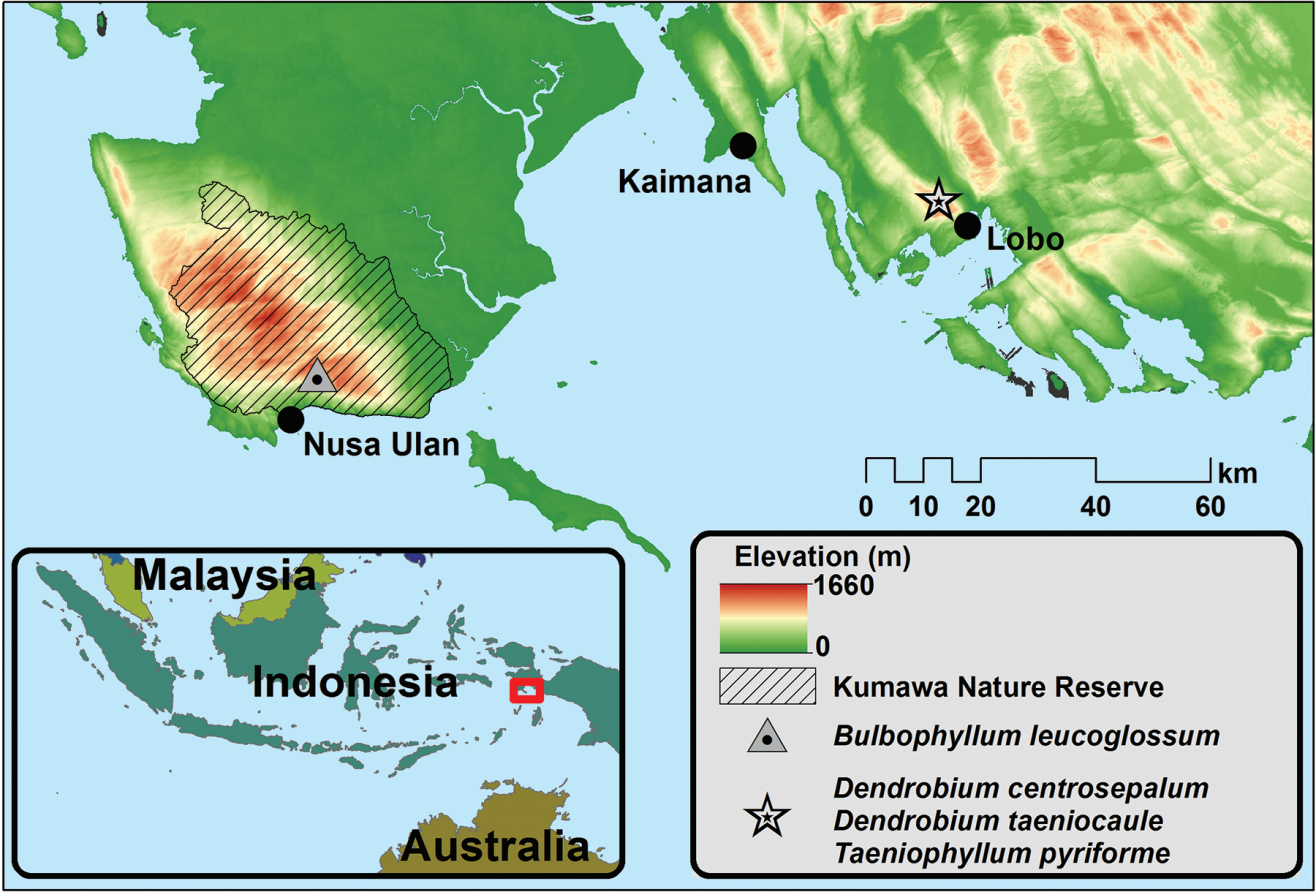
Main localities sampled during Lengguru 2014 expedition and distribution of the four new species in West Papua, Indonesia.

During the Lengguru expedition, we sampled 26 different sites in the limestone karst landscape (primarily swamp forest, mature lowland forest, alluvial forest, and submontane forest) from the sea shore to the top of the anticlines at around 1500 m elevation, in three main localities: Lobo village, Triton Bay; Urisa village, Arguni Bay; and Nusa Ulan, Kumawa Forest Reserve (Fig. 1). We gathered 72 fertile/flowering orchid specimens and associated material (pictures, silica-gel samples). We also collected living orchid specimens, which are now being cultivated in the Kebun Raya, Bogor (West Java) and in the Wamena Biological Gardens (Papua). Detailed examinations and comparisons with nomenclatural types of related species present in New Guinea, allowed us to identify four new species in the genera *Bulbophyllum* Thouars, *Dendrobium* Sw., and *Taeniophyllum* Blume. These novelties are described in the present paper, complemented with line drawings, photos, and information on habitats and distribution. As far as we now know, all four new species are endemic to the submontane forests of the Lengguru fold belt, at elevations above 1000 m.

## Materials and methods

Most orchid collections from the Lengguru expedition were preserved in Copenhagen mix (ethanol 70% and 5% glycerol). Specimens are deposited mainly in BO, with some duplicates in MAN, K, P and L (herbarium acronyms according to Thiers, continuously updated). Measurement and drawing of vegetative and reproductive parts were made on liquid preserved specimens, using a Wild Heerbrugg Switzerland Type 308700 stereo microscope. Additional data such as colour, habitat or ecology are mainly derived from field notes and high resolution pictures taken during the expedition.

## Taxonomic novelties

### Bulbophyllum (section Codonosiphon Schltr.) leucoglossum

Taxon classificationPlantaeAsparagalesOrchidaceae

Schuit., Juswara & Droissart sp. nov.

urn:lsid:ipni.org:names:77153386-1

Figs 2A , 3A–D

#### Diagnosis.

Similar to *Bulbophyllum pyroglossum* Schuit. & de Vogel because of the hinged lip and the hair-like appendages on the surface of the lip, but in that species the appendages are about four times longer, and they are discrete, subulate projections arranged in rows, not forming densely lacerate-fimbriate keels as in *Bulbophyllum leucoglossum*. Moreover, *Bulbophyllum leucoglossum* has two lamellae on the abaxial (concave) side of the lip; these are lacking in *Bulbophyllum pyroglossum*. The latter also has much broader and shorter petals, and an orange instead of a white lip.

#### Type.

Indonesia, West Papua Province, Kaimana Regency, Nusa Ulan village, Pegunungan Kumawa Nature Reserve, 04°0.3121'S, 133°5.1227'E, 15/11/2014, *Droissart & Juswara 1789* (holotype: BO!, spirit material).

#### Description.

Epiphytic *herb*. *Rhizome* short, creeping; *roots* wiry, branching, 0.5 mm diam. *Pseudobulbs* closely spaced, light green, narrowly ovoid, 2.0–2.4 × 0.7–0.8 cm, with c. 10 longitudinal grooves, 1-leaved. *Leaf* deeper green, linear-elliptic, gradually narrowed towards the base, 8.8–10.3 × 1.3–1.4 cm, thin-coriaceous, apex acute. Inflorescences arising from the base of the pseudobulb, becoming fascicled, erect, 1-flowered. *Peduncle* wiry, erect-patent, 1-flowered, 7–9 cm long, glabrous, with two tubular, 4 mm long peduncle-scales. *Floral bract* tubular, strongly oblique, 4.5 mm long, apex acuminate. *Pedicel-with-ovary* terete, very slender, weakly 6-ribbed, almost straight or curved, c. 2.5 cm long, glabrous. *Flowers* opening widely, the sepals patent to reflexed; sepals and petals maroon; lip white, at base wine-red, basal part of the keels sulphur-yellow; column cream-colour tinged maroon, swollen basal part and foot light green; anther pale greenish. *Dorsal sepal* linear-oblong, 14.3 × 2.8 mm, 3-veined, apex acute. *Lateral sepals* free, obliquely linear-oblong, 13.4 × 3.3 mm, 3-veined, apex acute. *Petals* linear-oblong, slightly widened towards the base, glabrous, 2.6 × 0.8 mm, apex obtuse. *Lip* clawed, slightly mobile, attached to the column-foot by a 0.3 mm long, 0.4 mm wide ligament; claw in the basal half almost quadrangular, tapering towards the blade, 2.4 × 1.5 mm, glabrous, with erect, hemi-elliptic, lobe-like margins in the basal half; blade narrowly oblong, slightly tapering towards the apex, cucullate, strongly convex above, 9.7 × 2.3 mm, margins deflexed, finely lacerate-fimbriate; blade adaxially with one median keel and two lateral keels on each side, the keels finely lacerate-fimbriate; on the concave abaxial side with two lacerate-fimbriate lamellae; apex obtuse. *Column* 2.3 mm long, curved, strongly swollen at the base, with a short but distinct, thick, 1 mm long column-foot; apical column-wings each with two short obtuse teeth, the wings 0.4 mm wide; *stigma* in lateral view with protruding lower margin; *anther* helmet-shaped, 0.6 mm long, very slightly papillose; *pollinia* not seen.

#### Distribution and habitat.

*Bulbophyllum leucoglossum* is only known from the Lengguru fold belt in West Papua. It is currently recorded from a single location in the Kumawa Forest Reserve, near the village of Nusa Ulan (Fig. 1). The only population seen so far was found in submontane forest at 1005 m elevation, the plants growing epiphytically about 1.5 m from the ground on a slender, moss-grown, overhanging tree trunk in medium-sloping terrain. More than ten individuals were observed in the collecting locality, but only one was flowering at the time of our fieldwork (November).

#### Etymology.

From the Greek *leuco*-, white, and *glossum*, tongue, referring to the largely pure white lip.

#### Notes.

A distinctive species because of the five laciniate-fimbriate keels on the lip, which give it a hairy appearance. The only other known species in the large section *Codonosiphon* with a distinctly hairy-looking lip is *Bulbophyllum pyroglossum* Schuit. & de Vogel from Papua New Guinea, which is similar in plant habit and in the size of the flower. See the diagnosis for the main differences between the two species.

### Dendrobium (section Calyptrochilus Schltr.) centrosepalum

Taxon classificationPlantaeAsparagalesOrchidaceae

Schuit., Juswara & Droissart sp. nov.

urn:lsid:ipni.org:names:77153387-1

Figs 2B , 3E–H

#### Diagnosis.

The short and dense inflorescences with small, purple flowers and green-tipped, long-apiculate sepals resemble those of *Dendrobium purpureum* Roxb., a lowland species from Maluku and Sulawesi. However, the plant habit of the latter is completely different, as *Dendrobium purpureum* has robust, many-leaved, cane-like, tufted stems up to more than 50 cm long. Vegetatively, *Dendrobium centrosepalum* is more similar to *Dendrobium aurantiroseum* P.Royen ex T.M.Reeve from New Guinea, which also has unifoliate pseudobulbs on a creeping rhizome. However, the latter is a species from high elevations (2100–3350 m) with pink flowers that are about twice as large, while the sepals are not apiculate; in addition, the cross-ridge on the lip is situated below the middle in *Dendrobium aurantiroseum* and above the middle in *Dendrobium centrosepalum*.

#### Type.

Indonesia, West Papua Province, Kaimana Regency, Lobo village, Triton Bay, 03°43.7962'S, 134°3.5962'E, 28/10/2014, *Droissart & Juswara 1736* (holotype: BO!, spirit material).

#### Description.

Epiphytic *herb*. *Rhizome* creeping, c. 3 cm long, growing downwards; *roots* 0.5 mm diam. *Pseudobulbs* closely spaced, erect, green tinged purplish, oblongoid-fusiform, 1.3–1.5 × 0.4 cm; main internodes 3; irregularly 5-ribbed; 1-leaved at apex, sometimes with a reduced additional leaf. *Leaves* glaucous green, deciduous, erect, narrowly elliptic, 3.3 × 1.1 cm; apex obtuse, minutely 3-dentate; margin smooth, slightly erose at apex; sheath very short. *Inflorescence* arising laterally from the upper internode of the leafless pseudobulb, erect, c. 12 mm long, c. 7-flowered; peduncle 5.2 mm long, covered by a few short scales in the basal part; rachis straight, 7 mm long. *Floral bracts* triangular, patent, 4.8 × 2.8 mm, apex acuminate, 3-nerved, glabrous. *Pedicel-with-ovary* narrowly clavate, c. 8.4 mm long, curved, with 5 rounded ribs, minutely papillose. *Flower* 10.5 mm long; sepals bright purple with greenish mucro; petals, lip and ovary bright purple. Sepals glabrous, but abaxially finely papillose on the slightly raised midvein; distinctly sharply apiculate at apex. *Dorsal sepal* ovate-oblong, 3.5 × 1.9 mm, 3-nerved; mucro 0.4 mm long. *Lateral sepals* obliquely narrowly ovate-oblong, much elongated in basal part, in total 10.8 × 2.8 mm, 4-nerved; mucro 0.9 mm long; mentum narrowly conical-cylindrical, 5.7 mm long, apex rounded, the closed apical part 4.8 mm long. *Petals* elliptic, 2.9 × 1.7 mm, emarginate, very shortly mucronate, 3-nerved, margin in upper half finely papillose. *Lip* when flattened subspathulate, 8.5 × 2.3 mm, at 5 mm above the base with a V-shaped transverse ridge, margins of the basal part adnate to the column-foot for 2.1 mm; apical part broadly elliptic, finely papillose along apical margin, apex rounded, minutely apiculate. *Column* rectangular, 1.9 mm long, wings truncate; foot 5.7 mm long; *stigma* semiorbicular, 0.8 mm wide, rostellum swollen, transversely oblongoid; *anther* cucullate-rectangular, 0.9 × 1.0 mm, minutely papillose, at base retuse, apex truncate and minutely erose; *pollinia* 0.7 mm long.

**Figure 2.**
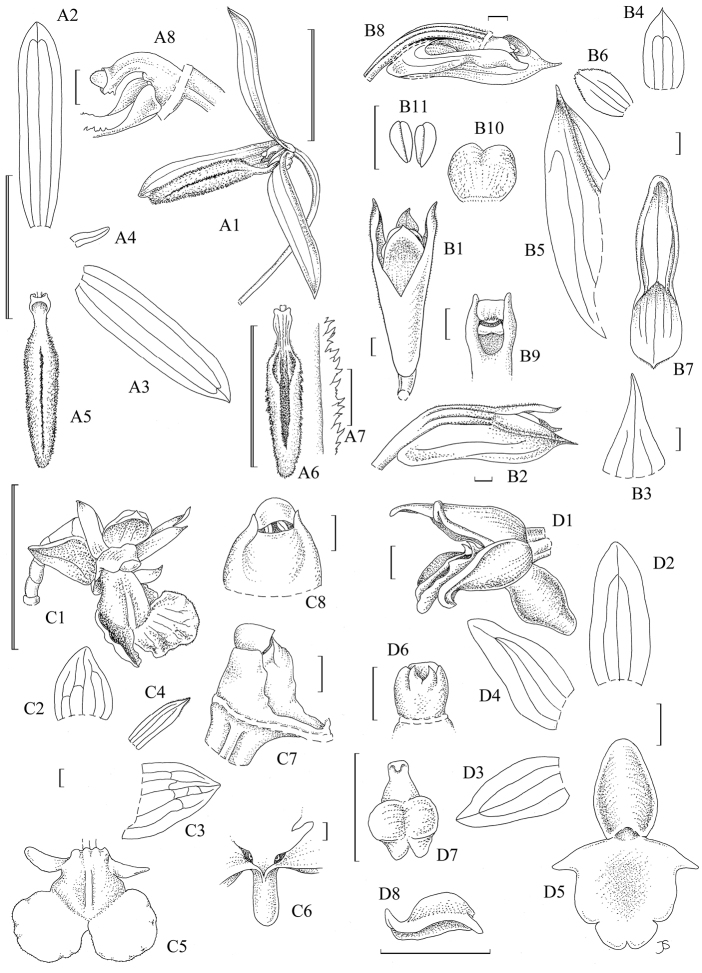
**A** *Bulbophyllum leucoglossum*: **1** flower **2** dorsal sepal **3** lateral sepal **4** petal; **5**, lip, adaxial view **6** lip, abaxial view **7** detail of crest on lip **8** column and base of lip; all after *Droissart & Juswara 1789*; **B** *Dendrobium centrosepalum*: **1** flower **2** flower, lateral view **3** floral bract **4** dorsal sepal **5** lateral sepal **6** petal **7** lip **8** flower, cut open **9** column **10** anther **11** pollinia; all after *Droissart & Juswara 1736*; **C** *Dendrobium taeniocaule*: **1** flower **2** dorsal sepal **3** lateral sepal **4** petal **5** lip **6** mentum **7** column, lateral view **8** column, ventral view; all after *Droissart & Juswara 1739*; **D** *Taeniophyllum pyriforme*: **1** flower **2** dorsal sepal **3** lateral sepal **4** petal **5** lip **6** column **7** anther, dorsal view **8** anther, lateral view; all after *Droissart &* Juswara 1735. Single scale bar = 1 mm; double scale bar = 1 cm. Drawing: Judi Stone.

**Figure 3.**
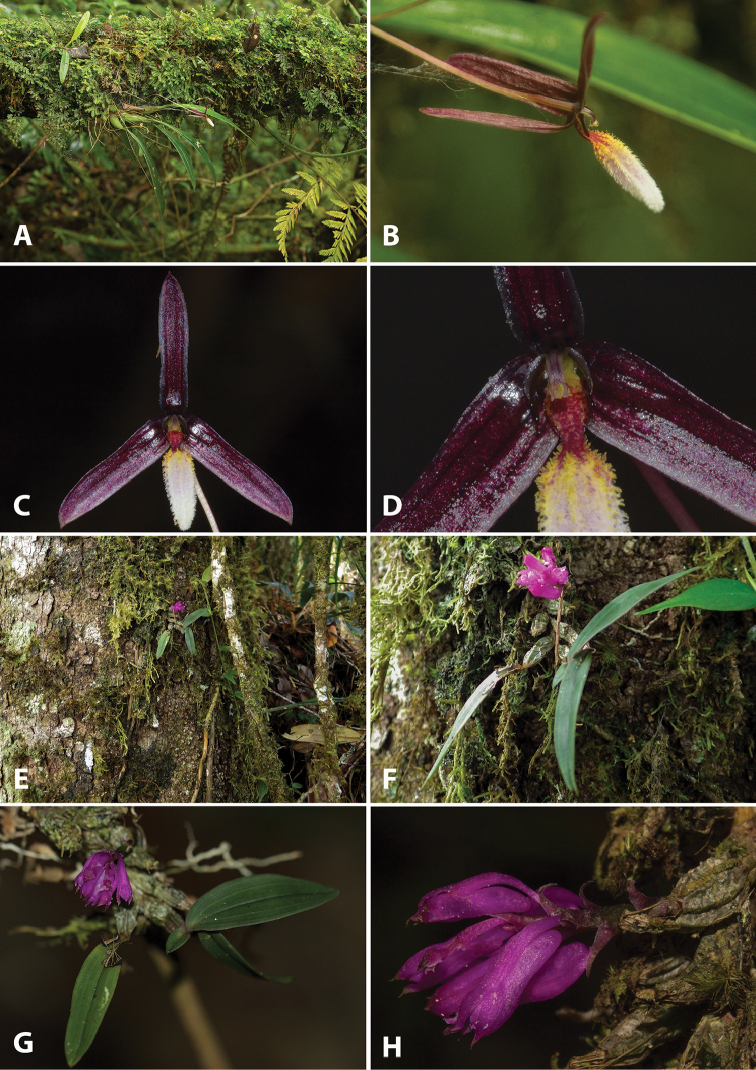
Photographs of living type specimens and habitats. *Bulbophyllum leucoglossum*: **A** habitat and habit **B** flower, side view **C** flower, front view **D** Flower close-up, showing details of the column and the labellum. *Dendrobium centrosepalum*: **E** habitat and habit **F, G** plant and inflorescence **H** inflorescence and flowers close-up. Photos: Vincent Droissart.

#### Distribution and habitat.

*Dendrobium centrosepalum* is only known from the Lengguru fold belt in West Papua. It is currently known from a single locality in the Triton Bay area, near the village of Lobo (Fig. 1). The only population seen so far was found in submontane forest at 1114 m elevation, the plants growing epiphytically on a thick, vertical, moss-and-lichen-covered trunk of a tree.

**Figure 4.**
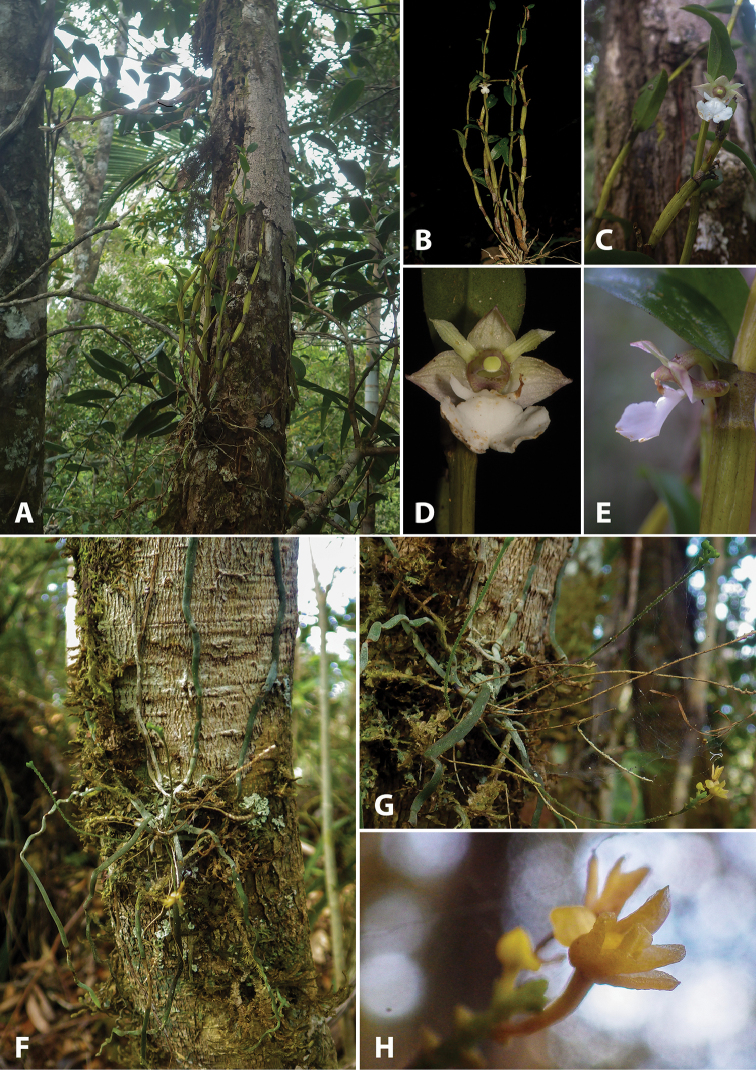
Photographs of living type specimens and habitats. *Dendrobium taeniocaule*: **A** habitat and habit **B** plant **C** flower and part of pseudobulb **D** flower, front view **E** flower, side view. *Taeniophyllum pyriforme*: **F** habitat and habit **G** plant and inflorescence **H** inflorescence and flowers close-up. Photos: Vincent Droissart.

#### Etymology.

From the Greek *centron*, a sharp point, referring to the apiculate sepals.

#### Notes.

The small, bright purple flowers in short and dense inflorescences superficially resemble those of such species as *Dendrobium dichaeoides* Schltr. and *Dendrobium limpidum* Schuit. & de Vogel, but these are quite different vegetatively, having elongate, leafy stems; in addition, in these two species the sepals are not sharply apiculate. See the diagnosis for additional comparisons.

### Dendrobium (section Brevisaccata Kraenzl.) taeniocaule

Taxon classificationPlantaeAsparagalesOrchidaceae

Schuit., Juswara & Droissart sp. nov.

urn:lsid:ipni.org:names:77153388-1

Figs 2C , 4A–E

#### Diagnosis.

Similar to *Dendrobium viridiflorum* F.M.Bailey in the flattened stems and the sympodially branching inflorescences with 1-flowered branches. The new species differs from *Dendrobium viridiflorum* in the relatively much shorter mentum (3.6 mm versus 7 mm long, with the free part of the lip being of about equal length in the two species), the relatively much broader dorsal sepal, in the lip being wider than long (versus longer than wide), and especially in the much wider (8.5 versus 3.2 mm), bilobulate (versus entire) mid-lobe of the lip.

#### Type.

Indonesia, West Papua Province, Kaimana Regency, Lobo village, Triton Bay, 03°43.7962'S, 134°3.5962'E, 28/10/2014, *Droissart & Juswara 1739* (holotype: BO!, spirit material).

#### Description.

Epiphytic *herb*. *Rhizome* short, creeping; *roots* 1 mm diam., minutely verrucose. *Pseudobulbs* erect, yellowish green, elongate, bilaterally flattened, 16–21 × 0.4–0.5 cm; internodes 2.3–2.6 cm long, each internode narrowed towards the base; leafy throughout, except for two or three basal internodes; 6–8-leaved. *Leaves* deep green, long-lived, patent, oblong, 2.0–3.3 × 0.6–0.9 cm; apex unequally bilobed; sheath much shorter than the internode. *Inflorescence* arising from the apex of the internodes in the upper part of the stem, sympodially branching with age, with up to 3 branches, each branch 3–8 mm long, 1-flowered; peduncle c. 6 mm long, covered with scales in the basal part. *Floral bract* triangular, closely appressed, 2.9 mm long, apex apiculate. *Pedicel* 2.6 mm long ovary at right angles to the pedicel, 2.6 mm long, glabrous; at the base of the pedicel with an abortive rachis. *Flower* 10.3 mm high, glabrous; sepals greenish white, tinged pale purple abaxially and on the mentum; petals pale green; lip white; column pale green suffused with purple; anther pale green. *Dorsal sepal* broadly ovate, 4.1 × 3.3 mm, 5-nerved, obtuse. *Lateral sepals* obliquely broadly ovate, the free part 5.3 × 4.3 mm, 5-nerved, obtuse; mentum cylindrical, 3.6 mm long, apex rounded, the closed apical part 2.8 mm long. *Petals* linear-oblong, 4.4 × 1.2 mm, acuminate, 3-nerved, margin in upper third finely papillose. *Lip* 3-lobed, clawed, when flattened 6.7 × 8.5 mm excluding the c. 4 mm long, linear claw; lateral lobes subfalcate-oblong, patent, 2.7 × 1.0 mm; mid-lobe clawed, bilobulate (the whole lip appearing 4-lobulate), 5.7 × 8.5 mm; mid-lobe claw obtrapeziform, 1.7 × 4.4. mm; lobules of the mid-lobe rectangular-orbicular, 3.3 × 4.2 mm; the whole of the lip, except for the lateral lobes and the lobules of the mid-lobe, occupied by a sharply delimited swelling, which has two broad longitudinal crests with a narrow rib in between and which projects backwards over the claw of the lip for 0.3 mm. *Column* broadly conical in front view, 1.7 mm long, wings very short, truncate; foot 3.6 mm long; *stigma* large, 1.6 mm wide, rostellum a simple transverse ridge; *anther* cucullate-rectangular, 1.1 mm wide, glabrous; *pollinia* not seen.

#### Distribution and habitat.

*Dendrobium taeniocaule* is only known from the Lengguru fold belt in West Papua. It is currently recorded from a single locality in the Triton Bay area, near the village of Lobo (Fig. 1). The only population seen so far was found in submontane forest at 1114 m elevation, the plants growing epiphytically on a vertical, lichen-covered trunk of a tree.

#### Etymology.

From the Greek *taenia*, a band or strap, and *caulon*, stem; referring to the flattened, band-shaped pseudobulbs.

#### Notes.

This species has only one obvious close relative, which is the widespread *Dendrobium viridiflorum*. Uniquely in section *Brevisaccata*, these two species share flattened stems and abbreviated inflorescences that produce up to 3, 1-flowered branches in succession over a longer period. The other species in the section have terete stems and flowers produced simultaneously on elongate racemes. In addition to clear morphological differences, as indicated in the diagnosis, the two first-mentioned species also have different ecologies. While *Dendrobium viridiflorum* is exclusively found in mangroves and coastal forest below 200 m, *Dendrobium taeniocaule* occurs in submontane forest above 1000 m.

### Taeniophyllum (section Loboglossum Schltr.) pyriforme

Taxon classificationPlantaeAsparagalesOrchidaceae

Schuit., Juswara & Droissart sp. nov.

urn:lsid:ipni.org:names:77153389-1

Figs 2D , 4F–H

#### Diagnosis.

Differs from all known species in sect. *Loboglossum* by the finely muricate peduncle and the hook-like basal lobules of the lateral lobes of the lip.

#### Type.

Indonesia, West Papua Province, Kaimana Regency, Lobo village, Triton Bay, 03°43.7962'S, 134°3.5962'E, 28/10/2014, *Droissart & Juswara 1735* (holotype: BO!, spirit material).

#### Description.

Leafless epiphytic *herb*. *Stem* very short; *roots* spreading, green, flattened, not branching, up to at least 35 cm long, 1.5–3 mm wide; some of the roots closely appressed to the bark of the phorophyte, others free hanging. *Inflorescences* c. 3 producing flowers at the same time, suberect, 5–6 cm long; peduncle filiform, 0.5 mm diam., rather sparsely muricate with c. 0.3 mm long projections; near the middle with a very small peduncle-scale; rachis distichous, glabrous, up to c. 16-flowered, with the flowers opening in succession, one or two at a time, gradually elongating, up to 11–14 mm long. *Floral bracts* cupular, in lateral view triangular, subacute, 1.2 mm long; successive bracts on the same side of the rachis 1.8 mm apart. *Flowers* apparently non-resupinate (always?), c. 5 mm high including the spur, glabrous, pale brownish yellow. *Dorsal sepal* ovate, 3.3 × 1.5 mm, obtuse, 3-veined. *Lateral sepals* somewhat obliquely ovate, 2.6 × 1.3 mm, obtuse; 3-veined; abaxially at the apex with a short lamella along the midvein. *Petals* obliquely ovate, 3.2 × 1.5 mm, obtuse, 3-veined. *Lip* spurred, 3-lobed, ecallose, margins erect to incurved, when flattened 2.7 × 3.2 mm; lateral lobes semi-oblong, rounded, 2.0 mm long from base of lip to base of mid-lobe, at the base with an erect, narrowly triangular-uncinate lobule; mid-lobe reniform, 0.7 × 1.2 mm, emarginate. *Column* short, cylindrical, 1.1 long, 1.0 mm wide, with a short, bidentate rostellum; *stigma* shallowly concave; *anther* cucullate, 0.9 cm long, 0.6 cm wide, apex rostrate, recurved; *pollinia* not seen.

#### Distribution and habitat.

*Taeniophyllum pyriforme* is only known from Papua. It is recorded from a single locality near the village of Lobo in the Triton Bay (Fig. 1). The only population seen so far was found in submontane forest at 1114 m elevation, the plants growing epiphytically on a sparsely moss-covered tree trunk at 1.5 m above the ground.

#### Etymology.

From the Latin *pyriforme*, pear-shaped, referring to the shape of the spur.

#### Notes.

This inconspicuous but distinctive species would seem to fit best in section *Loboglossum*, on account of the clearly lobed lip, elongate inflorescence, distichous rachis, and glabrous ovary. However, a muricate inflorescence has not been reported for this section before (although the otherwise very different *Taeniophyllum toranum* J.J.Sm. is described as having a furfuraceous-punctate peduncle and rachis, while the no less distinct *Taeniophyllum pulvinatum* is said to have a minutely glandulose peduncle). The basal, hook-like lobules on the lip are also unique.

It is likely that a large number of species of *Taeniophyllum* still await discovery in New Guinea. We believe this to be the case because most of the species are easily overlooked; the flowers often last only a day or less; and many of the known species are only recorded from the type. New Guinea is clearly the centre of diversity for this genus, with 130 species currently recorded.

## Conclusions

The four new species described here demonstrate that, most likely, many new species still await discovery in poorly explored parts of New Guinea. Among our collections from the Lengguru area, 1 out of 8 species of *Bulbophyllum*, 2 of 22 *Dendrobium* species, and 1 of 2 *Taeniophyllum* species proved to be new to science, and 3 of these novelties were collected on the same day near Lobo village. This only represents species found in flower during our expedition, which is certainly a minority of the total orchid flora of the area.

The new species of *Bulbophyllum* and *Dendrobium* described here, while distinctive, do not present striking new features for these genera; they are easily classified among the known species. The new *Taeniophyllum*, on the other hand, appears to be without any obvious close relatives. This genus in particular warrants much more attention from scientific collectors and taxonomists; the diversity and phylogenetics of this genus are still poorly understood.

## Supplementary Material

XML Treatment for Bulbophyllum (section Codonosiphon Schltr.) leucoglossum

XML Treatment for Dendrobium (section Calyptrochilus Schltr.) centrosepalum

XML Treatment for Dendrobium (section Brevisaccata Kraenzl.) taeniocaule

XML Treatment for Taeniophyllum (section Loboglossum Schltr.) pyriforme
